# Binocular vision and the control of foot placement during walking in natural terrain

**DOI:** 10.1038/s41598-021-99846-0

**Published:** 2021-10-22

**Authors:** Kathryn Bonnen, Jonathan S. Matthis, Agostino Gibaldi, Martin S. Banks, Dennis M. Levi, Mary Hayhoe

**Affiliations:** 1 Department of Neural Science, New York University, New York, USA; 2 Department of Psychology, University of Texas at Austin, Austin, USA; 3 Department of Biology, Northeastern University, Boston, USA; 4 School of Optometry, University of California, Berkeley, Berkeley, USA

**Keywords:** Sensorimotor processing, Visual system, Vision disorders, Human behaviour

## Abstract

Coordination between visual and motor processes is critical for the selection of stable footholds when walking in uneven terrains. While recent work (Matthis et al. in Curr Biol 8(28):1224–1233, 2018) demonstrates a tight link between gaze (visual) and gait (motor), it remains unclear which aspects of visual information play a role in this visuomotor control loop, and how the loss of this information affects that relationship. Here we examine the role of binocular information in the visuomotor control of walking over complex terrain. We recorded eye and body movements while normally-sighted participants walked over terrains of varying difficulty, with intact vision or with vision in one eye blurred to disrupt binocular vision. Gaze strategy was highly sensitive to the complexity of the terrain, with more fixations dedicated to foothold selection as the terrain became more difficult. The primary effect of increased sensory uncertainty due to disrupted binocular vision was a small bias in gaze towards closer footholds, indicating greater pressure on the visuomotor control process. Participants with binocular vision losses due to developmental disorders (i.e., amblyopia, strabismus), who have had the opportunity to develop alternative strategies, also biased their gaze towards closer footholds. Across all participants, we observed a relationship between an individual’s typical level of binocular visual function and the degree to which gaze is shifted toward the body. Thus the gaze–gait relationship is sensitive to the level of sensory uncertainty, and deficits in binocular visual function (whether transient or long-standing) have systematic effects on gaze strategy in complex terrains. We conclude that binocular vision provides useful information for locating footholds during locomotion. Furthermore, we have demonstrated that combined eye/body tracking in natural environments can be used to provide a more detailed understanding of the impact of a type of vision loss on the visuomotor control process of walking, a vital everyday task.

## Introduction

The task of walking in the natural world is a visuomotor control problem. Walking over irregular terrain relies on the ability to efficiently gather information about the upcoming path in order to guide foot placement. In flat terrains where the choice of footholds are not heavily constrained by the environment, humans typically select a preferred gait cycle, defined by step length, width, and duration. The preferred gait minimizes the energetic cost by exploiting the passive physical dynamics of the body^1–3^. However, in rough and unpredictable terrain, the walker must make a trade-off between the energetic efficiency of the preferred gait cycle and the need to place the feet in stable locations to support continuous locomotion. Controlled laboratory experiments directly examining vision and gait have established that walkers require information from around 2 steps ahead in order to successfully navigate collections of obstacles or targets along a smooth path^4–11^. Recent work in natural environments suggests that the demands of finding stable footholds are met by devoting gaze to the region 2–3 footholds ahead, and slowing down to allow time to visually locate a suitable foothold^12^. Because information from two footholds ahead is necessary in order to take advantage of the passive dynamics of the body, this allows walkers to compromise between the energetic optimum and locating stable footholds. Thus, given the environment, walkers actively modulate their gaze behavior to gather visual information from the upcoming terrain that will result in foothold choices that best support their gait.

While it is clear that visual information processing is critical to the task of walking in complex terrains, very little is known about the nature of the visual information that is used to locate footholds and more generally to support locomotor control. Almost all the behavioral work on visual control of locomotion has been done in laboratory settings with smooth walking surfaces, with a single or small number of planar obstacles or targets^13–16^. Consequently, essentially nothing is known about the visual search process that locates footholds during locomotion in more complex terrains. Since humans take a step every 400–500 ms, they have only a limited time to identify a suitable place to put the foot. As a result, only 2–3 fixations are possible per step so the search process must be efficient. It may be the case that simple spatial features such as large light patches, indicating convex surfaces are sufficient. However, a defining feature of irregular terrain is depth variation in the walking surface. These variations require regulation of the descending step so that the foot makes contact with the appropriate forces for that height. Thus the search process must identify a relatively smooth surface within stepping range, *and* know its three-dimensional location. It is in this context that the extraction of depth information is likely to be important. Since the walker is in constant motion, motion parallax cues could provide sufficient information. However, binocular depth perception provides higher precision depth information than motion parallax^17^. Studies of binocular disparity information report supra-threshold disparities in natural scenes during indoor and outdoor walking^18^, even at fixation distances greater than 15 m^19^. Interestingly, McCann et al.^20^ showed that binocular thresholds remain lower than monocular thresholds up to this distance, which is well beyond the range most important for selecting footholds. Thus, impairing binocular vision effectively removes some of the visual information typically available during walking.

Existing laboratory studies of participants with experimentally-imposed deficits to binocular vision, either as a result of occlusion, or blurring of vision in one eye, have demonstrated that it decreases performance in stereopsis^21,22^ and related tasks, like eye hand coordination(Refs.^23,24^; see^25^ for a comprehensive review). More recent laboratory studies of walking and impaired binocular vision have noted that participants exhibit slower walking and raise the foot higher when stepping up to or over an obstacle^15,26–28^. This indicates that binocular vision plays a role in visual control of foot placement, with impairment of binocular vision leading to an increase in the safety margin for foot height. When the step height becomes familiar with repetition of the task, the safety margin in foot height is reduced, suggesting that locomotor behavior is adaptive. Buckley et al.^29^ investigated individuals with developmentally-related reductions in stereoacuity and found a similar increased toe height when stepping over obstacles, despite the fact that these individuals had the opportunity to adapt to their reduced stereoacuity. Thus it appears binocular depth perception plays a role in the visuomotor control of walking at least in the case of stepping over obstacles. However, because these studies did not track eye movements, they are not informative about the relation between gaze and control of gait. A study by Hayhoe et al.^30^ tracked gaze in monocular and binocular vision while participants walked over obstacles, and found, similar to the studies described above, that participants raised the foot higher when stepping over the obstacles. In addition, they found that gaze location stayed on the obstacle for a longer period as the foot neared the obstacle, indicating that the gaze–gait relationship is affected when one eye is occluded.

To shed light on how footholds are selected in natural environments, and more generally, how increased uncertainty affects the visuomotor control process in locomotion, we manipulated the availability of binocular information during locomotion in an unconstrained outdoor environment, while tracking eye and body movements of normally-sighted participants. We also included a small number of participants with long-standing deficits to their binocular vision (resulting from amblyopia and/or strabismus) to gain further insight into how deficits in binocular vision impact the visuomotor control process during walking. Developmental visual disorders (e.g., amblyopia, strabismus) affect approximately 5% of the population and lead to deficits in binocular vision. By including these individuals, we are able to examine the walking performance of individuals given their typical vision across a range of levels of binocular visual function. Furthermore, because these individuals have had the opportunity to adapt to the reduction in binocular information, they may have learned to make greater use of other types of information (e.g., motion parallax). Walkers generally attempt to minimize energetic costs in the context of other constraints, and it is also possible that participants with long-standing impairments to binocular vision have learnt different trade-offs between sensory and motor uncertainty than individuals whose binocular vision was only temporarily impaired.

## Results

**Figure 1:**
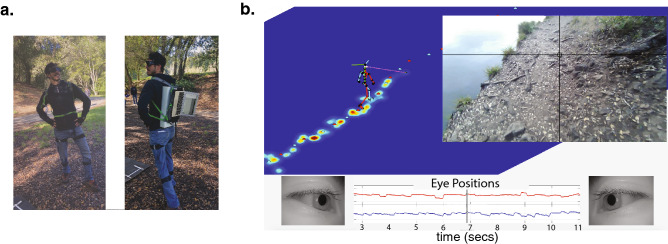
(**a**) A participant wearing the Pupil Labs binocular eye tracker and Motion Shadow motion capture system with the data recording computer on the participant’s back. Optometrist sunglasses were used to shade the eyes to improve eye tracker performance. (**b**) A sample frame from of the data from Supplementary Video 1. On the right is the view of the scene from the head camera, with gaze location indicated by the cross-hair. Below are the horizontal and vertical eye-in-head records. The high velocity regions (steep upward slope) show the saccades to the next fixation point, and the lower velocity segments (shallow downward slope) show the slower eye movement that stabilizes gaze on a particular location in the scene as the participant moves towards it, resulting a characteristic saw-tooth appearance for the eye signal. On the left, the stick figure shows the skeleton figure reconstructed from the Motion Shadow data. This is integrated with the eye signal which is shown by the blue and pink lines. Gaze location history is indicated by the Gaussian heat maps. The blue and red dots show the foot plants recorded by the motion capture system.

This study aimed to examine the effects of increased uncertainty on the visuomotor control processes involved in walking, by manipulating the availability of binocular information. Participants were directed to walk along a trail with varied terrain while we measured their eye and body movements. The total distance of the walk was approximately 1 mile. Figure 1a shows an author wearing a Pupil Labs eye tracker (Pupil Labs, Berlin, Germany) and a Motion Shadow motion-capture suit (Motion Workshop, Seattle, USA) in the environment where the data were collected. Participants completed the walk twice. During one of the walks we impaired participants’ binocular vision by blurring the input to one eye using a Bangerter Occlusion Foil (Fresnel Prism and Lens Co., Bloomington, MN, USA; see Table 1 for the effects on individual participants’ monocular and binocular vision). In the analyses that follow, we: (a) establish how patterns of gaze and walking behavior change across varied terrains (verifying findings first reported in^12^ in a different unconstrained environment); and (b) compare aspects of the participants’ gaze and walking behaviors across these two conditions (Binocular vs. Blur) to determine how the increase in uncertainty in the visual signal (i.e., impairment to binocular vision) affects the visuomotor control process during walking. We also collected and analyzed data from 4 participants with binocular visual disorders using only the protocol for the binocular condition. We include these participants in the analyses only where explicitly specified.

**Table 1:** Participant visual and demographic characteristics—normally sighted individuals.

	P1	P2	P3	P4	P5	P6	P7	P8
Age	27	39	34	26	29	24	24	54
Gender	M	M	M	F	F	F	F	M
Height (cm)	183	193	183	165	157	163	168	178
Leg length (cm)	103.5	101	96.5	89	81	85	90	96.5
Binocular acuity (logMAR)	0.04	0.42	− 0.08	0.26	− 0.12	0.18	− 0.06	0.1
Binocular acuity w/ left blurred (logMAR)	− 0.02	0.64	− 0.04	0.5	− 0.18	0.12	− 0.04	0.52
Monocular (L) acuity (logMAR)	0.14	0.54	− 0.14	0.44	− 0.16	0.4	0.02	0.12
Monocular (L) acuity blurred (logMAR)	0.66	0.8	0.7	0.78	0.56	0.68	0.6	0.58
Randot $\textregistered$ (arcsecs)	40	20	50	25	20	70	30	50
Randot$\textregistered$ w/left blurred (arcsecs)	70	200	> 400	70	50	140	70	> 400
First condition	Bino.	Blur	Bino.	Blur	Bino.	Blur	Bino.	Blur
Eye dominance	R	R	R	R	L	R	R	L
Leg dominance	R	R	L	R	R	L	L	R
Y-balance composite (% of leg length)	64.57	101.24	83.07	73.50	95.47	83.82	69	87.91

Figure 1b shows a sample frame of Supplementary Video 1, which provides a visualization of the data collected over the course of the experiment. This visualization shows the gaze location both in the video image and relative to the body as it moves through the world via the moving stick figure on the left (see “Methods” section). On the left, gaze locations are indicated by the Gaussian heat maps and the sequence of foothold locations are indicated by the red and cyan dots. The gaze vectors for the right and left eyes are shown by the pink and blue lines. Gaze behavior was similar to that described in^12^ where participants make high velocity saccadic eye movements from one location to the next along the future travel path, and then stabilizing eye movements (i.e., vestibular ocular reflex, optokinetic nystagmus, and/or smooth pursuit) counter-rotate the eyes to maintains a stable image as the head moves forward. This is indicated by the characteristic saw-tooth pattern shown at the bottom of Fig. 1, where the saccades are indicated by the abrupt shifts in eye-in-head position, and the stabilizing eye movements are indicated by the slow change as the eye counter-rotates to maintain gaze position during the forward movement.

### Gaze distributions vary with terrain

**Figure 2:**
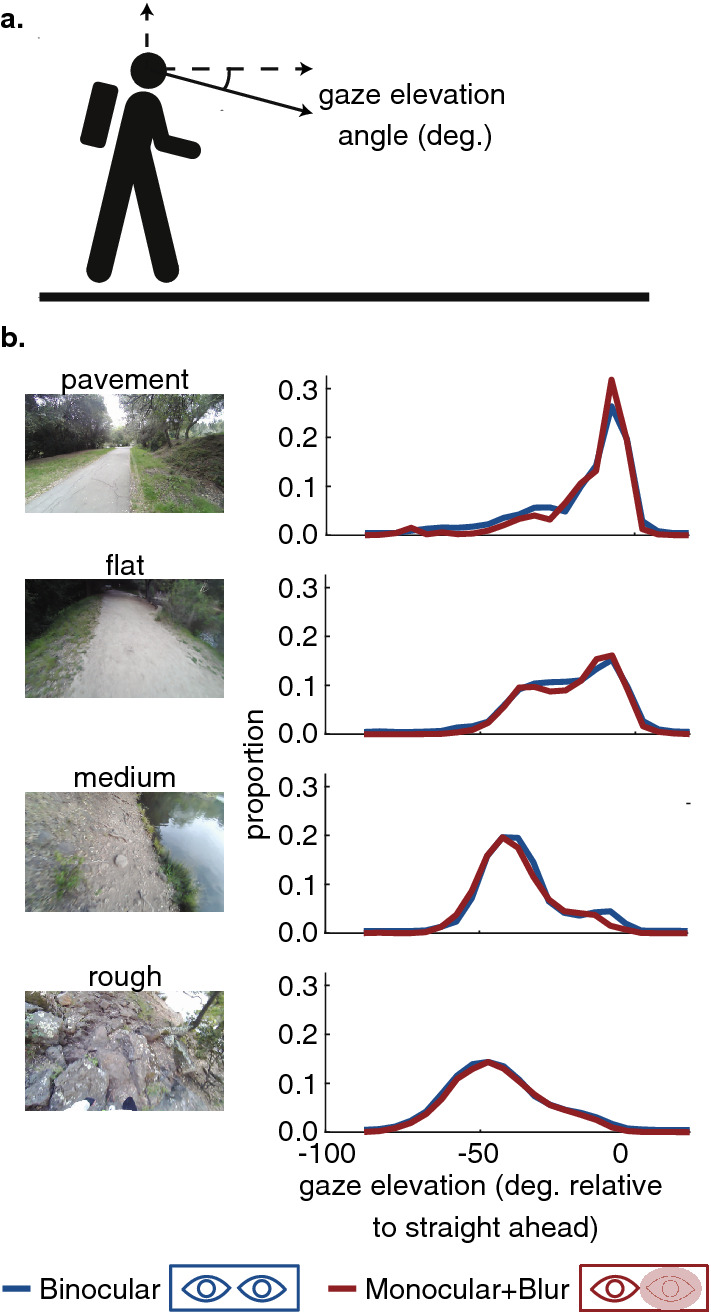
Analysis of gaze distributions do not show consistent differences in gaze distributions across the binocular and blur conditions. (**a**) Schematic showing how the gaze elevation angle is measured (where the vertical axis is defined by gravity). (**b**) Distribution of gaze angles relative to the horizontal for the different terrain types. Gaze angles between $-\,40^{\circ }$ and $-\,50^{\circ }$ are roughly 2–3 footholds ahead. The blue line shows data for normal binocular vision and red shows the distributions in the stereo-impaired condition where one eye was blurred using a a 0.2 Bangerter foil. Data are pooled across 8 participants with stereoacuity in the normal range.

As reported in^12^, gaze behavior varies with the terrain. Gaze is directed mostly near the body in rough terrain in order to guide foothold selection, and at more distant locations (off the ground) in smoother terrain. This is shown in Fig. 2, which plots the distribution of gaze angles, for the different types of terrain. In the plot, zero indicates gaze at a distant location, parallel to the ground plane. Gaze angles around $-\,45^{\circ }$ are closer to the walker, when gaze is allocated to the ground near 2–3 footholds ahead. The distributions of gaze angles shift drastically across the terrains. Even the paved road and the flat dirt path lead to distinctly different distributions, suggesting different demands on vision. This might be a consequence of the different frictional properties of the surfaces, requiring slightly different forces at contact, or perhaps the result of different expectations about the properties of the surface. In the rough terrain, gaze density is centered on an elevation of approximately $-\,45^{\circ }$ from the horizontal, reflecting the need to locate stable footholds. Generally speaking, as the demands of the terrain increase, more of the gaze time is dedicated to elevations close to $-\,45^{\circ }$ that indicate the observer is looking at the ground.

The red distributions in Fig. 2 show the blur condition, where vision in one eye was blurred. The distributions are very similar in the two conditions. Using a within-subjects comparison, we compared participants’ median gaze angle and found no significant differences (Wilcoxon signed-rank test; $p=0.25$, $p=0.84$, $p=0.64$, $p=1.00$ for the pavement, flat, medium, and rough terrains respectively). To examine whether there were consistent changes to the distributions not well captured by a shift in the central tendency, we performed two-sample Kolmogorov–Smirnov tests comparing the gaze elevation distributions on a per-participant basis. These tests did show a difference in the gaze elevation distributions between the two conditions for all participants across all terrains. However, in all cases the shifts were small and inconsistent in direction across participants.

### Gaze allocation to upcoming footholds is terrain-dependent

**Figure 3:**
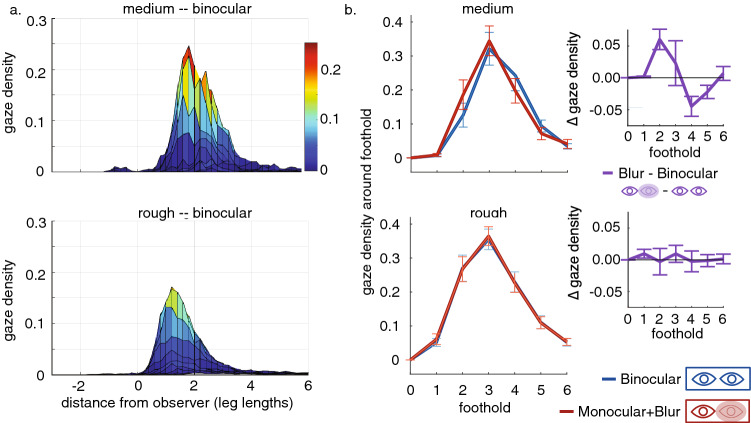
An analysis of gaze densities demonstrates a bias in gaze toward the body for participants during the blur condition in the medium terrain. (**a**) The average gaze distribution relative to the planted foot in the binocular condition for the medium (top) and rough (bottom) terrains. This distribution is two-dimensional, though here it is viewed from a single angle that allows us to view the distribution along the path, i.e., the x-axis here corresponds to the participant’s current walking direction so that the positive direction is down the path in front of the observer. The relationship between these distributions and the summary statistics shown in b, are described in the “Methods” section and follow the methodology depicted in Fig. 6. (**b**) The two panels on the left show gaze density around footholds 1–6 footholds ahead, derived as described in the “Methods” section, for medium (top) and rough terrains (bottom). Note that the rough terrain shows a greater allocation of gaze to nearer footholds (specifically 2 footholds ahead). The blue and red curves show the binocular and blur conditions for the 8 normal participants. The panels on the right show the within participants differences (purple) between the binocular and blur conditions. Error bars are $\pm \,1$ SEM.

To examine in more detail the relationship between gaze and foothold selection in the medium and rough terrains, we calculated the distribution of fixation locations on the ground plane, relative to the planted foot (e.g., Fig. 3a shows a sideview of these distributions for the binocular condition). One of the challenges in comparing these gaze distributions across conditions is that the variability in the path taken by the walker adds variability to the gaze distribution. Since participants paths are not perfectly straight, and indeed are quite irregular in rough terrain, the gaze distribution relative to the planted foot is smeared out by these path variations. In order to address this challenge^12^, we developed a method for computing the gaze density around upcoming footholds (see also the “Methods” section), examining gaze distributions after subtracting off upcoming footholds. A diagram of this analysis is depicted in Fig. 6 in the “Methods” section. By recalculating the gaze distributions around upcoming footholds and summing the gaze density near those footholds, we are able to measure the gaze allocated to particular footholds in each condition. Following this methodology, we show the average gaze densities around upcoming footholds in Fig. 3b for the medium and rough terrains. Note that for both the medium and rough terrains that gaze is allocated near footholds 2–4, with more gaze allocated to the second foothold in the rough terrain (particularly in the binocular condition).

### Gaze allocated closer to the body with diminished binocular vision

#### Binocular versus blur conditions

Figure 3b compares the binocular and blur conditions for the medium and rough terrains. In the medium terrain participants in the Blur condition show gaze locations shifted toward slightly closer footholds compared to the Binocular condition (see the within participant comparison depicted by the purple curve). Although the shift is small, it appears to be reliable. In the rough terrain, gaze density still peaks at the second foothold, but shifts away from foothold four and more density is allocated to the second foothold. In this terrain there are no differences between binocular and blur conditions.

#### Developmental losses

**Figure 4:**
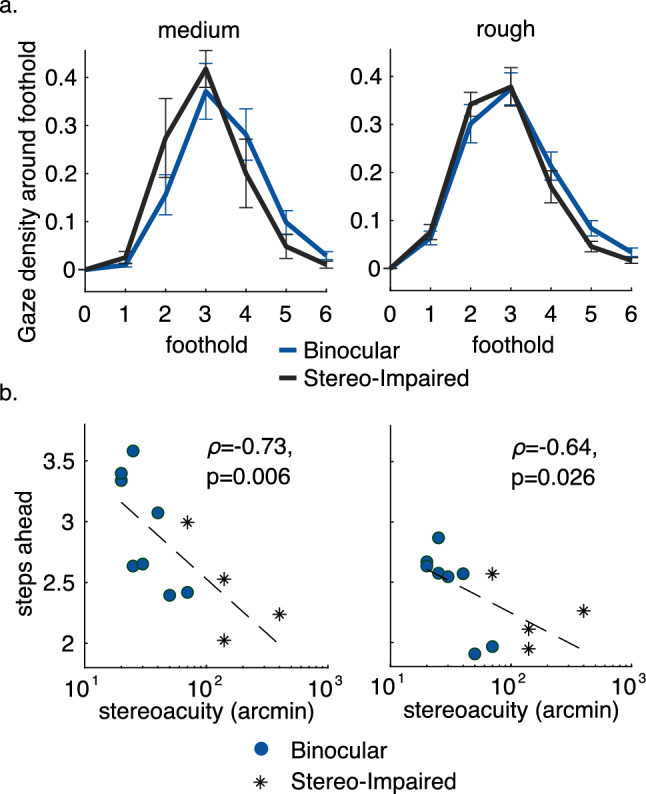
Examining the original 8 participants as well as an additional 4 participants with impairment to binocular function due to visual disorders reveals a relationship between the bias of gaze toward the body and stereoacuity. (**a**) Gaze density around footholds as in Fig. 3b, showing the binocular condition from the original participants and stereo-impaired participants, for medium and rough terrains. Error bars are $\pm \,1$ SEM between participants. The dark blue curve shows the binocular conditions from Fig. 3b, and the light blue curve shows data for the 4 stereo-impaired participants. (**b**) Average footholds ahead as a function of participant stereoacuity. Average footholds ahead (y-axis) is calculated by taking the vector average of the gaze density around the foothold plotted in a (above). Stereo-acuity (x-axis) is measured as described in the Methods using the Randot metric. Correlation for the linear regression fits are shown in the Figure, with associated significance values.

The within-participant comparison between the binocular and blur conditions (with and without a blurring filter over one eye) allow removal of between participant variation, but has the disadvantage that these participants are unaccustomed to the loss of binocular visual function imposed by the blur in one eye. This might induce transient effects potentially unrelated to stereo impairment, such as binocular rivalry or reduction of the field of view. We evaluated performance in the normally and stereo-impaired groups (both walking with binocular vision). Figure 4a shows the distribution of gaze relative to different footholds, as in Fig. 3b, for the normally sighted group in the Binocular condition and the stereo-impaired group. Although there is substantial variability between participants, the stereo-impaired participants appear to shift their gaze to closer footholds. This appears more marked in the medium terrain, as in Fig. 3b. Since the small number of participants in the stereo-impaired group makes statistical comparisons somewhat tenuous, we present this data in a different way, examining the relationship between participant stereo-acuity and the gaze–gait relationship during walking. Because of Despite the classification of the participants into normal and stereo-impaired groups, stereo-acuity varied considerably between the 8 participants in the binocular vision condition. Figure 4b shows the relation between gaze and foot placement as a function of stereo-acuity, measured for individuals in each group. The data in Fig. 4 demonstrate clearly that participants move their fixations closer to their body with decreasing stereoacuity.

### Walking speeds vary with terrain (but not with binocular information)

**Figure 5:**
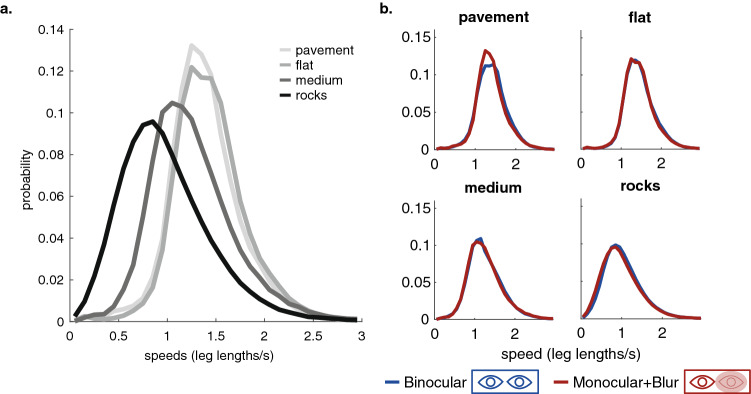
Walking speeds are slower in rough terrains but there is not a consistent difference in walking speeds across the binocular and blur conditions. (**a**) Walking speed distributions for the different terrains measured in leg length/s. (**b**) Walking speed distributions for normal (blue) and blur (red) conditions, for each of the terrain types.

Changes in the terrain also lead participants to change their walking speed. In the rougher terrain they slow down to about 0.8 m/s, from about 1.4 m/s on the pavement, as shown in Fig. 5a. A similar finding was also reported in^12^ and is possibly limited by the time taken to visually locate a suitable foothold. Reduced walking speed reflects a deviation from the energetically optimal preferred gait determined by the passive dynamics^1,3,31^ but it seems likely that the added energetic cost is small and less important than the need for stable footholds. Thus the precise linkage between vision and the control of stepping is highly sensitive to properties of the terrain and the corresponding shifts in the demands placed on vision. Figure 5b shows the speed distributions for the binocular versus the blur condition for each of the terrains. As with gaze angle distributions, walking speed distributions are very similar. We found no significant differences in the median walking speeds when compared on a within participants basis (Wilcoxon signed-rank test; $p=0.84$, $p=0.55$, $p=0.25$, $p=0.08$ for the pavement, flat, medium, and rough terrains respectively. We do note that that these data suggest a small, but non-significant slowing of the median walking speed in rough terrain). To examine whether there were consistent changes to the distributions of walking speeds not well captured by a shift in the central tendency, we performed two-sample Kolmogorov–Smirnov tests comparing the walking speed distributions on a per-participant basis. The two-tail Kolgomorov–Smirnov tests did show differences in the walking speed distributions between the two conditions for participants in all terrains. However, in all cases the shifts were small and inconsistent across participants.

## Discussion

Visual guidance of locomotion in the natural environment is a complex sensorimotor control problem that is influenced by a number of interacting factors. The nature of the terrain itself has a dominant effect on the properties of the sensorimotor control loop for walking. Even subtle differences such as that between a paved road and a flat dirt path affect the allocation of gaze to far versus near targets. With the paved road, participants can use global information about the path to program the footsteps and little visual information is needed. The dirt path (flat terrain) introduces some added uncertainty, possibly related to the programming of the forces appropriate for a surface with different friction or even as a result of a different prior for this kind of path. As the demand for visual monitoring increases in more complex terrains, participants slow down, as was observed in^12^. This suggests that the acquisition of visual information may be rate limiting, although we must also consider the possibility that participants reduce speed because the rough terrain makes the execution of the steps more difficult or that it increases their safety during walking. As a result, it is not clear whether this slowing in more difficult terrains results primarily from sensory, motor, or criterion-related factors.

Unlike changes to terrain complexity, deficits to binocular depth perception do not appear to influence walking speed, suggesting that it is not a rate-limiting factor in the same way. However, we do observe: (1) a shift in gaze allocation to closer footholds in medium terrain during temporary deficits to binocular vision and; (2) a relationship between stereoacuity (a measure of binocular depth perception) and gaze allocation across upcoming footholds. With reduced stereoacuity, more gaze was allocated to footholds closer to the body in the medium terrain. Looking closer to the body results in a closer fixation, which increases the magnitude of the disparity^18^, and specifically for a depth discontinuity. It also reduces the time between acquisition of the visual signal and the execution of the action, a period where information loss presumably increases with time. This result is comparable to the results of Hayhoe et al.^30^, where participants kept gaze on an obstacle until the body was closer in conditions when one eye was occluded. There is very little effect of impairment to binocular depth perception in the flat terrain, as might be expected from the reduced need for visual information to locate footholds. The strongest effect of binocular depth perception is observed in the medium terrain where we see both a shift in gaze allocation toward the body in the blur condition for participants with experimentally impaired depth perception (Fig. 3b, top panel) and a negative relation between between stereoacuity and the average gaze location (Fig. 4b, left panel). These effects are diminished (or absent) in the rough terrain. We do not observe a shift in gaze toward the body in the blur condition (Fig. 3b, top panel), though we do still observe a negative relationship between between stereoacuity and the average gaze location (Fig. 4b, right panel). The decreased (or absent) gaze shift is likely because there is a limit on the benefits of shifting gaze closer to the body. Previous work has indicated that for walking, foothold planning, and obstacle avoidance, humans need to have information from approximately 2 steps ahead^8,11^. This means that there is a limit on the benefits of shifting gaze closer to the body. It is likely that in the rough terrain, participants are approaching that limit. Thus we can no longer detect a shift in their gaze toward the body, because that is a less optimal strategy with which to deal with the increased uncertainty.

Even in challenging environments, the primary effect appears to be the location of gaze relative to the body, with other aspects of the sensorimotor control of locomotion (e.g., walking speed, fixation durations) being comparable. Given the findings illustrated in Figs. 3 and 4 , we expected that the tendency to fixate closer to the body would show up as a difference between gaze angle distributions in Fig. 2. It seems likely that this effect is quite subtle and is simply not pronounced enough to show up in the distributions shown in Fig. 2. Thus the primary driver of gaze behavior is the terrain, with the more subtle effects of binocular visual impairment appearing in detailed analyses of the gaze–gait relationship. In this respect our results are a little different from previous studies that demonstrated an increase in foot height when stepping over obstacles^28^, since the primary effect of reduced stereo-acuity was on gaze location and information acquisition, rather than on locomotion itself.

Visual uncertainty also affects walking performance. In our study, we found that increased uncertainty (long-standing or temporary impairment to binocular vision) resulted in participants allocating their gaze closer to their body. Dominguez-Zamora et al.^32^ investigated the effects of visual uncertainty on walking in the laboratory by varying the contrast of patches projected on the floor. Participants were required to step on a sequence of three targets. There were a number of differences in their findings, as compared with ours. They found that increased uncertainty led to increased fixation durations as well as larger foot placement errors. Longer fixations linked to the step suggest greater uncertainty in the information acquired during the fixation. In our study, the distributions of fixation durations are not effected by manipulations of both terrain and binocular vision. Dominguez et al.^32^ also reported that increased uncertainty, participants increased the time interval between the gaze and the body, i.e., that participants looked farther ahead. This effect is in the opposite direction from what was observed here. However, the average look-ahead times (and corresponding look-ahead distances) in that study were much shorter than we observed (maximum 0.5 s vs. 1–2.5 s), and became even shorter as participants progressed through the 3-step sequence in the experiment, to the point where participants were looking at the current foothold at the end of the sequence. Thus the restriction to a 3 step sequence leads to a rather different pattern of gaze–gait timing likely due to the limited length of the path. Thus clear effects of sensory uncertainty were observed in their study, but visuomotor coordination for their task was rather different from normal outdoor walking, like that which we observe in our study.

In summary, it is clear that binocular depth cues are one of the sources of information that can be used in locating footholds when walking in uneven terrain. Deficits (imposed or long-standing) to binocular visual function resulted in a measurable change in participant’s gaze–gait strategy, though it did not severely disrupt the participant’s ability to walk along the path. This utility of binocular visual information and the presence of measurable adjustments to behavior given deficits to binocular visual function is consistent with earlier literature indicating that participants with deficits to binocular depth perception lift their feet higher to create greater safety margins when they are engaged in a task that requires them to step over obstacles^28–30^. During normal walking the safety margin for foot height may be as little as 1 or 2 cm. This corresponds roughly to a disparity of about 0.5–1 min arc, at a distance of 2 or 3 steps ahead, which should be above stereoacuity thresholds in fairly comparable real-world contexts^17,20^. Therefore, it is perhaps not surprising that binocular depth information is used by those with intact binocular vision to program foot placement in rough terrain. We also note that the such a cue may become even more important in other contexts, e.g., if participants are asked walk at a much faster pace. Stereopsis allows you to look further ahead, so that you don’t have to spend as much effort or time examining the region near your feet if you have good stereopsis. And if you were being chased by a bear, that may improve your chance of survival. Finally, and more generally, this work provides a step forward in examining how the visuomotor control process that governs walking in everyday life adapts or even deteriorates as vision declines. The use of simultaneous body and eye tracking in outdoor/everyday environments generates rich data that can be used to make detailed analyses of visuomotor control and truly examine the consequences of such changes for a person’s everyday life.

## Materials/methods

### Participants

A total of twelve participants completed the experiment. Written, informed consent was obtained for all participants in accordance with The University of California Berkeley Institutional Review Board. Participants were treated according to the principles set forth in the Declaration of Helsinki of the World Medical Association. These experiments were conducted after approval from the University of California Berkeley Institutional Review Board. Additionally, we received informed consent for publication of identifying information/images in an online open-access publication (see Fig. 1a). Eight of the participants reported corrected or corrected-to-normal vision, while four of the twelve participants had existing diagnoses of developmental visual disorders (i.e., amblyopia, strabismus; see Table 2). Participants had no known motor impairments.

**Table 2:** Participant visual and demographic characteristics—individuals with amblyopia (A) and/or strabismus (S).

	A1	A2	S1	S2
Non-dominant eye	L	L	L	R
Refractive correction (R)	$+\,5.50/-\,4.75$	$+0.50/-\,0.50$	–	$+\,1.00/-\,1.25$
	x 008	x 111		x 180
Refractive correction (L)	$+\,5.75/-\,5.75$	$+\,4.00/-\,1.00$	–	$+\,1.00/-\,1.25$
	x 172	x 030		x 180
Ocular alignment (prims diopters)—distance	Ortho	Ortho	4 LET	RET, 1
				Hypertropia
				> 45
Ocular alignment (prims diopters)—near	Ortho	Ortho	6 LET	RET, 1
				Hypertropia
				> 45
Age (years)	22	54	31	26
Gender	M	F	F	M
Height (cm)	178	163	175	185
Leg length (cm)	90	86	93	98
Binocular acuity (logMAR)	.18	.3	.02	.04
Monocular (L) acuity (logMAR)	.52	.86	.22	.06
Monocular (R) acuity (logMAR)	.22	.32	.02	.76
Randot$\textregistered$ (arcsecs)	140	140	70	> 400
Eye dominance	R	R	R	L
Leg dominance	R	R	R	R
Y-balance composite (% of leg length)	106.20	69.57	77.15	86.74

### Equipment

We measured the eye and body movements of participants walking along a hiking trail with terrain sections of varying complexity, relying on methods developed by Matthis et al.^12^. The eyes were tracked using a Pupil Labs binocular mobile eye tracker recording eye position at 120 Hz. The tracker’s scene camera recorded the scene from the participant’s point of view at 30 Hz. To allow the infrared cameras of the eye tracker to work in sunlight, participants wore standard dark filters used by optometrists following pupil dilation. The filters were fitted onto the eye tracker frame like sunglasses. The gaze was recorded in both eyes in the binocular condition but because of difficulties with the lens holder for the blur filter we were unable to record both eyes in the blur condition. Though both eyes were recorded in the binocular condition, we used monocular data for the analyses of both the binocular and blur conditions (choosing the same eye, the right eye, in both conditions).

Kinematics were recorded using the Motion Shadow full body motion capture system that has 30 inertial measurement units recording at 100 Hz at different body location. Data were recorded on a MacBook Air mounted on a lightweight backpack. Since both devices were collecting data on the same computer, we were able to sync these two data streams together in time using the shared computer timestamp. There was an average frame drop of $\sim \,2\%$. We used a polyphasic anti-aliasing filter to resample and temporally align the eye and body movement data (see https://www.mathworks.com/help/signal/ref/resample.html) at 120 Hz. The calibration section describes how we were able to align the data spatially. All post-processing analyses were performed offline using custom MATLAB code.

### Experimental task

Participants walked along a hiking trail continuously for about 10–15 min, out and back, stopping briefly to perform calibrations 3 times during the experiment. The overall distance walked was a little over a half mile. For the purpose of analysis and to compare to previous work^12^, parts of the trail were pre-designated as pavement, flat, medium or rough (see examples of terrain in Fig. 2). The path was selected to be relatively straight, relatively free from elevation gain, and to have identifiable segments of varying difficulty. Each of these sections were approximately 90 m, 200 m, 60 m, and 25 0m respectively. For reference, the length of a standard American football field is about 90 m and a football pitch is 100–110 m. The result is that even in the medium terrain (60 m) we have eye/body movement data from at least 100 steps.

The Pavement terrain condition was a straight paved path containing no obstacles. The Flat terrain condition was a packed earth trail containing very few obstacles. The Medium terrain consisted of tree roots, bark with a somewhat irregular surface and occasional embedded rocks. Visual guidance was needed to place the feet, but finding an available foothold was relatively easy (most ground locations were viable footholds). The Rough terrain consisted of large embedded rocks and surface irregularities. More substantial visual guidance was necessary to support locomotion in this terrain, as most ground locations were not viable footholds. The analyses in this paper focus largely on the Medium and Rough terrains. In a general sense, both of these terrains result in the walker allocating a sizable chunk of their gaze to the ground in order to visually guide/adjust their gait. The qualitative difference between these two terrains rests largely in the difference between the availability of viable footholds, with fewer viable footholds available in the Rough terrain. As a result, it may be appropriate to think of participants in the Medium terrain as performing obstacle avoidance while participants in the Rough terrain are actively search for specific footholds.

### Experimental conditions/groups

Eight normally-sighted participants performed the experiment twice, once with their existing vision (Binocular condition) and once with a 0.2 Bangerter foil over one eye to blur vision and thus impairing binocular vision. This is referred to as the Blur condition. The filter was designed to reduce acuity to approximately 0.7 LogMAR (approximately 20/100), though in practice the acuity varied some ($0.67\pm 0.09$ LogMAR). Table 1 contains the measures of acuity and stereoacuity across conditions for each participant. The order of binocular and blur conditions was counterbalanced across participants (Table 1 lists the first condition completed by each participant). Half of the stereo-impaired participants completed the binocular experiment twice and we took the second run for the analysis.

Four participants with developmental visual disorders associated with deficits to binocular vision and stereopsis completed the experiment once. We refer to this group as StereoImpaired. Of the 4 participants, two had strabismus (without associated monocular acuity loss), and two had anisometropic amblyopia (see Table 2 for additional information). Three of the participants (PD1-3) had participated in extensive stereopsis training (Ref.^33^, see also^34,35^) for the purpose of regaining some stereopsis and other binocular function.

### Experimental protocol

Prior to completing the walking task, participants completed a battery of acuity, stereoacuity, and motor tests and measurements (see Table 1). This included several measurements of visual acuity using a LogMAR chart, including binocular acuity (binocular condition), their binocular acuity with Bangerter Foil covering their left eye (blur condition), their monocular acuity (left) and their monocular acuity (left) with the eye covered by the Bangerter Foil. Measures of stereoacuity for both the binocular and blur conditions were taken using the Randot stereoacuity test (Stereo Optical, Inc., Chicago, IL). All eight participants that completed the blur condition had worse stereoacuity in the blur condition. Finally participants completed the Y-balance test^36^ and Table 1 reports the composite score in percent of leg length. This test measures dynamic balance and requires that participants balance on a planted foot while moving their other foot in one of three directions which form a Y: anterior (to the front), posterolateral (behind and to the side of the planted foot), posteromedial (behind and away from the planted foot), where the anterior direction is $135^{\circ }$ from the posteromedial and posterolateral directions and the posterolateral/posteromedial directions are separated by $90^{\circ }$. We also recorded participant height (cm), leg length (cm), dominant eye (L/R), and dominant leg (L/R).

Participants began the main experiment by putting on the equipment and completing the calibration protocol described below. They then walked along a paved section from the starting location to the beginning of the Flat terrain path. The experimenter followed the participant closely at all times. Following calibration, and also at the end of the walk, participants completed a validation task whereby they walked across a flat path that contained 6 brightly colored markers on the ground arranged 3 m apart in a straight line (e.g., Supplementary Video 2). participants were instructed to traverse the path of markers while always maintaining fixation on the nearest marker. This data task was later used to validate the calibration procedure for each participant as described in^12^.

### Calibration

The eye tracker recorded the participant’s point of regard (POR) in 2D pixel coordinates relative to the outward facing scene camera that was mounted on the participant’s head. The kinematic data from the inertial motion capture system recorded movement in millimeters relative to magnetic north (determined by the triaxial magnetometers in the IMU sensors) and the gravity vectors (determined by the IMU accelerometers). In order to calculate a participant’s 3D gaze vector, it was necessary to situate the 2D data from the eye tracker into 3D reference frame of the motion capture system. To do this, we used the calibration method described in^12^, that used the vestibular ocular reflex (VOR) to determine the mapping between the eye tracker POR data and the IMU coordinate system in order to recover participants’ 3D gaze vector relative to the body and world. Participants stood on a calibration mat that had marks for the participants’ feet, a high visibility marker located 1 m ahead of the vertical projection of the midpoint of the ankle joint, and a 0.5 m piece of tape at the same distance. Following the experimenter’s instruction, the participant maintained fixation on that point while slowly moving their head up/down, left/right, and along the diagonals. In addition to help determine the participant’s 3D gaze vector by relating eye and head movements, data from this portion of the record were used to calibrate the eye tracker (similar to the head tick method described in^37^, except that our participants moved their head smoothly). Details of calculation of the Participants’ 3D gaze, including estimation of the ground plane, are further elaborated in^12^. As described in that paper, the resulting calibration error is about $\pm \,1^{\circ }$ Other sources of error such as parallax error, IMU drift, and the flat ground plane assumption are also relatively modest and described in that paper.

### Gaze/gait analyses

We adopted a set of gaze/gait analyses first presented in^12^. The aim of these analyses is to describe the gaze allocated to upcoming footholds in a way that removes the variability introduced by non-straight walking paths. The method begins by situating participants’ gaze on the ground in a reference frame where the origin is their currently planted foot. We assume that the ground is a flat plane perpendicular to the direction of gravity at the same elevation as the planted foot. We split up each walk into individual steps. A step consists of the period between the heelstrike of a given foot and the sample before the heelstrike of the other foot. (That is, each step period began at the onset of the double support phase and continued until the end of the single support phase.) For each recorded gaze intersection with the ground, we subtracted the X, Y ground plane coordinates of the gaze intersection with the X, Y ground plane coordinates of the heel marker of the stepping foot. The resulting gaze coordinates are then in reference from with the location of their planted foot at the origin. We then repeat this reference frame calculation for the next 6 upcoming footholds. The procedure for this process was identical to the method used to align gaze to the planted foot, only now instead of subtracting the XY location of the planted foot from each gaze point, we subtracted the XY locations of the foothold location of the steps that were coming up in the future. In all we represented participants’ gaze in 7 different reference frames one centered on the planted foot (Foothold N), one centered on the first upcoming foothold (Foothold N + 1, or the destination of the currently swinging foot), and one each for Foothold N + 2, N + 3, and N + 4, N + 5, and N + 6.

**Figure 6:**
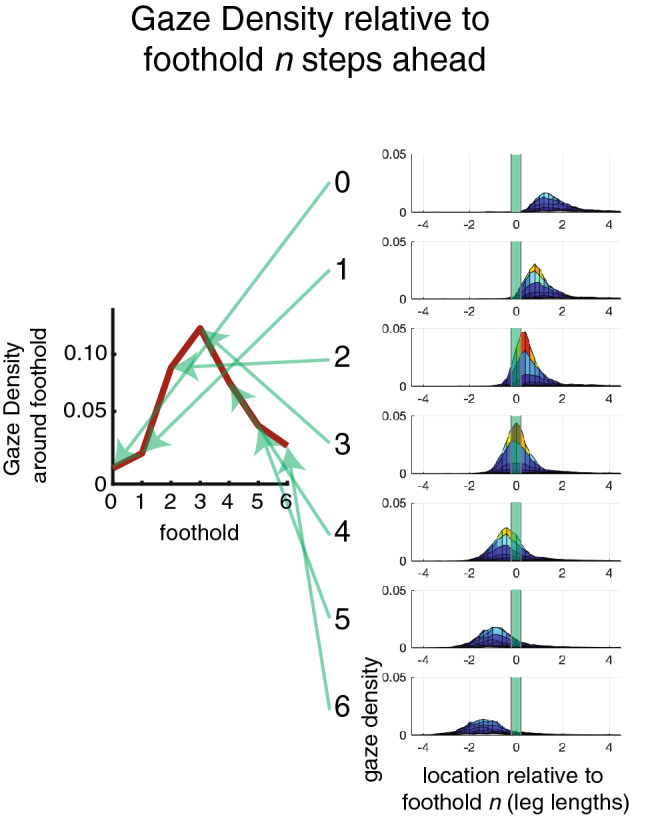
Calculating gaze density relative to nth foothold. The gaze density around upcoming footholds is the integral of the gaze density within 0.3 leg lengths of the origin in each foothold-centered reference frame (Bottom panels, numbered 0–6). The summary panel (Top) shows the gaze densities as a function of foothold and thus provides a relative measure of the gaze allocated to that foothold during walking in a particular condition.

Figure 6 shows how these data can be summarized to express how gaze is allocated to different footholds. The density plots along the bottom of the figure show the gaze distributions plotted in the various foothold reference frames. From each distribution, we sum the gaze density around the origin (foothold location) as a function of the foothold, where 0 is the planted foot and 1=6 are the next 6 footholds. Thus the top panel show the relative gaze allocation to upcoming footholds.

### Calculating gaze density

We calculated empirical gaze density distributions for each of the foothold-centered reference frames from the planted foot (N) to the 6th foothold ahead (N + 6). Note that the probability was normalized by the total number of recorded samples in each walk, which was larger than the number of gaze intersections with the ground plane (because not all of the participants’ gaze vectors intersected the ground plane). As a result, the sum of the total probability distribution in each reference frame was equal to the proportion of the time that participants spent looking at the ground.

To summarize the gaze density distribution, we calculated the gaze density around upcoming footholds as depicted in Fig. 6. This quantity is the integral of the gaze density within 0.3 leg lengths of the origin in each foothold-centered reference frame. This analysis shows the proportion of the time that participants spent looking at that upcoming foothold. In this manner we can determine where gaze is allocated on average during a bout of walking in a particular terrain.

## Supplementary information

Supplementary Information 1.

Supplementary Video 1.

Supplementary Video 2.
