# Supplementary material for: Binocular vision and the control of foot placement during walking in natural terrain
Source: Sci Rep. 2021 Oct 22;11:20881. doi: 10.1038/s41598-021-99846-0 (PMC8536664; doi:10.1038/s41598-021-99846-0)
Supplement: Supplementary file 1 — Supplementary Information 1. [file 41598_2021_99846_MOESM1_ESM.pdf]

Supporting Information Appendix (SI)

SI Movies

The supplementary videos are examples of the collected data from one participant.

Supplementary Video 1. Walking. This video contains examples from one participant of the data collected in the rough, medium and flat terrains. Each frame contains the following panels: (Right) The view of the scene from the head camera, with gaze location indicated by the cross-hair. (Below) The horizontal and vertical eye-in-head records. The high velocity regions (steep upward slope) show the saccades to the next fixation point, and the lower velocity segments (shallow downward slope) show the slower eye movement that stabilizes gaze on a particular location in the scene as the participant moves towards it, resulting a characteristic saw-tooth appearance for the eye signal. (Left) A view of the motion capture data. The stick figure shows the skeleton figure reconstructed from the Motion Shadow data. This is integrated with the eye signal which is shown by the blue and pink lines. Gaze location history is indicated by the Gaussian heat maps. The blue and red dots show the foot plants recorded by the motion capture system.

Supplementary Video 2. Validation task. During the validation task, participants walked across a flat path that contained 6 brightly colored markers on the ground arranged 3m apart in a straight line. They were instructed to walk from the first marker to the last, maintaining fixation on the nearest upcoming marker. Each frame of the video is laid out in the same manner as Supplementary Video 1.

Fixation Durations

Another aspect of performance that might reflect greater difficulty in finding stable foothold in rough terrain is the duration of the fixations that locate footholds. Gaze data were analyzed to extract the fixations as described in the Methods section, and fixation duration distributions are plotted in Supplementary Figure 1. The left plot shows fixation duration distributions for each terrain type for the binocular condition. Fixation duration clusters around 100-300 ms for all terrains and there is no significant effect of terrain (Kruskal-Wallis analysis of variance, $\chi^2 = .86$, $p = .84$). A similar set of distributions is observed when vision is stereo degraded and we found no significant difference in the median fixation duration for the binocular versus blur conditions (Wilcoxon signed-rank test; $p = .74$, $p = .62$, $p = .36$, $p = .75$ for the pavement, flat, medium, and rough terrains respectively.

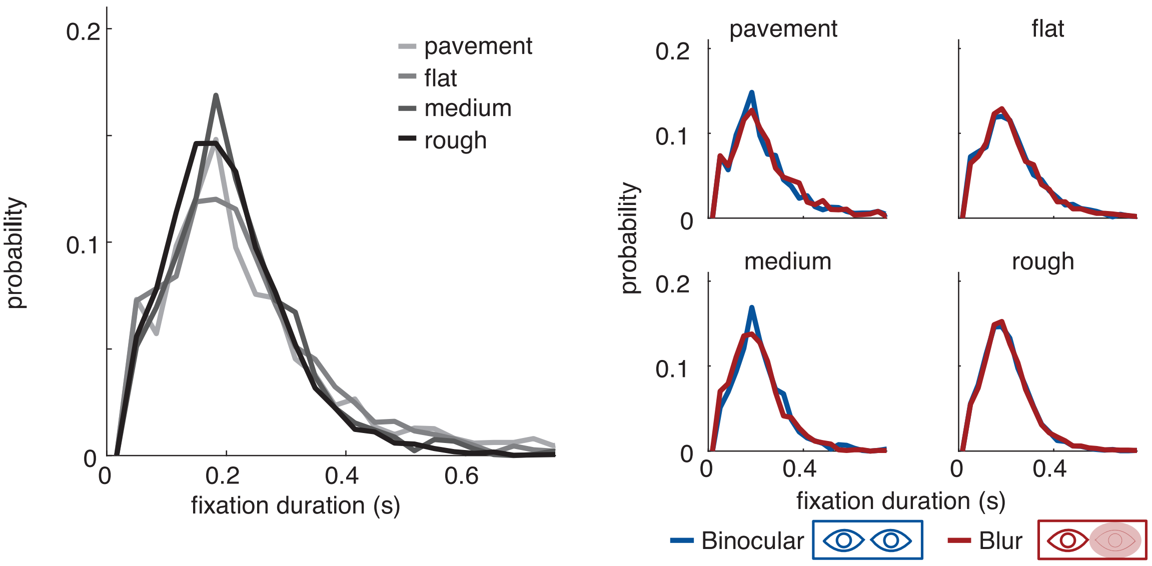

Figure 1: An analysis of fixation durations reveals very little variation across both terrain type and viewing condition. (a) Fixation duration distributions for the four terrains. Distributions on the left are for normal binocular vision. (b) The right panel shows a comparison of normal (blue) and blur (red) conditions for each terrain separately. There were no significant differences in the medians of the distributions.

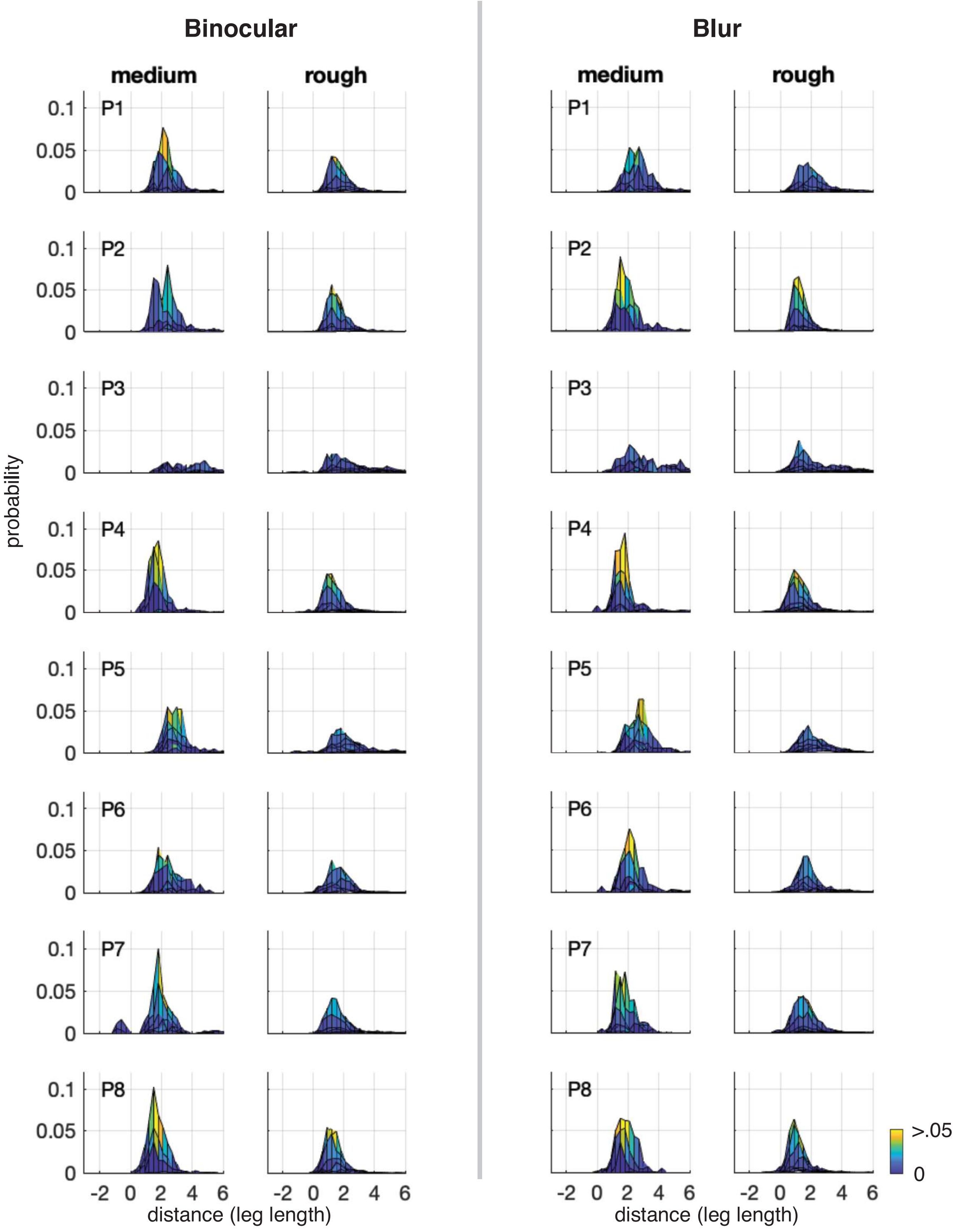

Figure 2: Gaze distributions for the medium and rough terrains for each participant in the Binocular (left) and Blur (right) conditions. Compare to Figure 3a

??

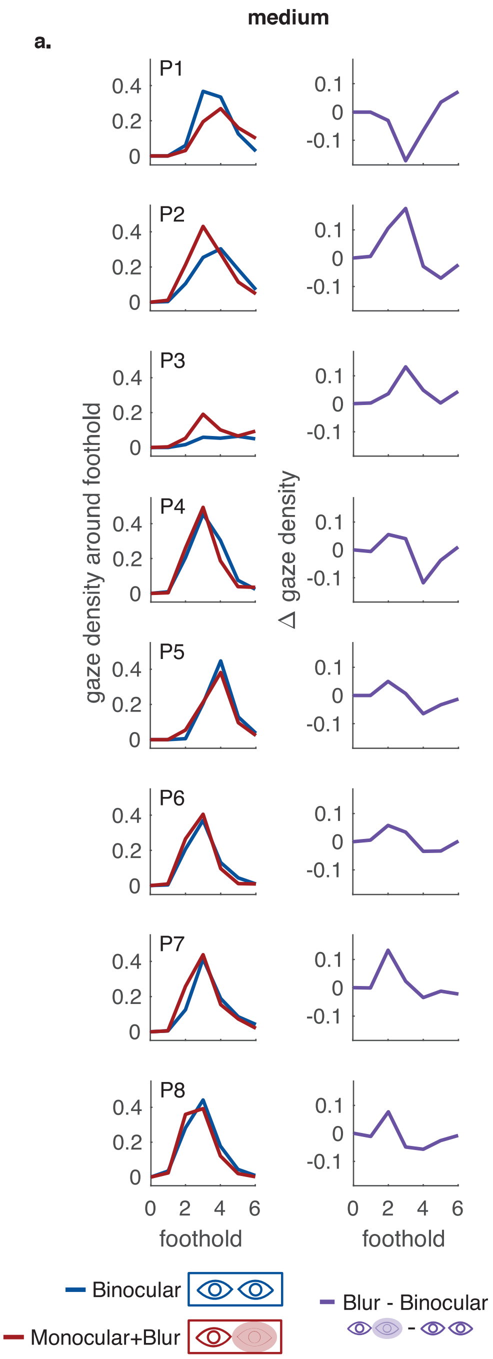

Figure 3: Gaze densities around upcoming footholds in the medium (a) and rough (b) terrain for each participant. Compare to Figure 3b

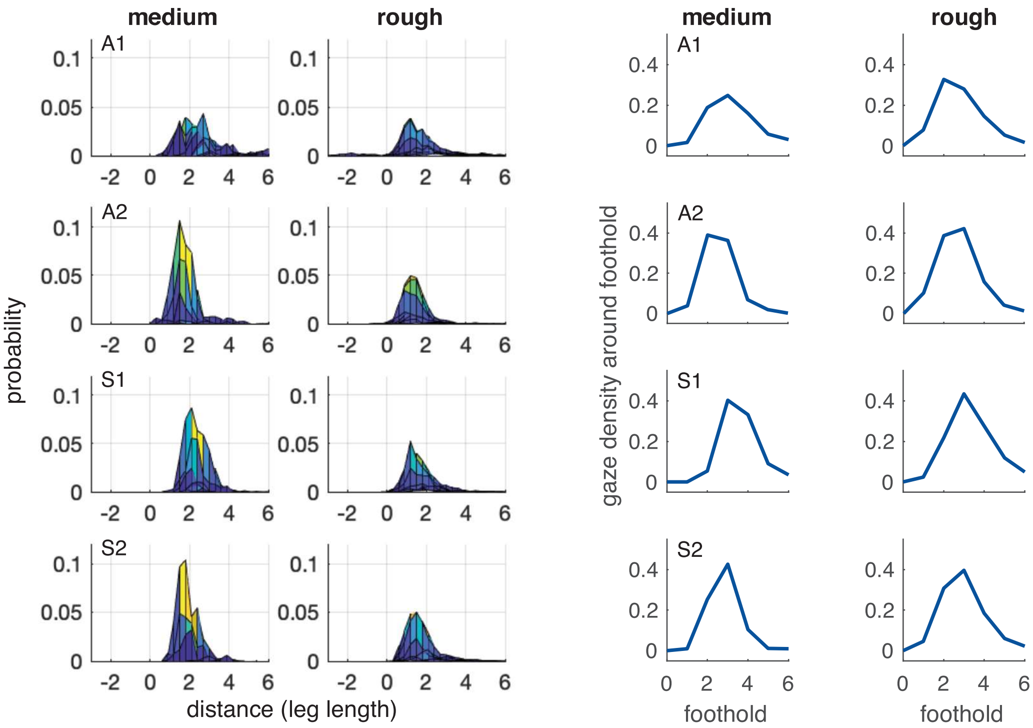

Figure 4: (a) Gaze distributions and (b) gaze densities around upcoming footholds for the medium/rough terrains for participants with binocular visual disorders. Compare to Figure 4.
